# Biomarkers of Brain Damage and Postoperative Cognitive Disorders in Orthopedic Patients: An Update

**DOI:** 10.1155/2015/402959

**Published:** 2015-08-31

**Authors:** Dariusz Tomaszewski

**Affiliations:** Department of Anesthesiology and Intensive Therapy, Military Institute of Medicine, Szaserow 128 Street, 04-141 Warsaw, Poland

## Abstract

The incidence of postoperative cognitive dysfunction (POCD) in orthopedic patients varies from 16% to 45%, although it can be as high as 72%. As a consequence, the hospitalization time of patients who developed POCD was longer, the outcome and quality of life were worsened, and prolonged medical and social assistance were necessary. In this review the short description of such biomarkers of brain damage as the S100B protein, NSE, GFAP, Tau protein, metalloproteinases, ubiquitin C terminal hydrolase, microtubule-associated protein, myelin basic protein, *α*-II spectrin breakdown products, and microRNA was made. The role of thromboembolic material in the development of cognitive decline was also discussed. Special attention was paid to optimization of surgical and anesthetic procedures in the prevention of postoperative cognitive decline.

## 1. Postoperative Cognitive Disorders: Terminology, Clinical Spectrum, Incidence, and Risk Factors

Postoperative cognitive disorders are common in elderly (>65-year-old) patients. Cognitive dysfunction is present more often in orthopedic patients than in any other hospitalized group. It includes the deterioration of perception, memory, information analysis, attentional focus, concentration, and patients' response [1]. Those disorders are divided into postoperative delirium, postoperative cognitive dysfunction (POCD), and dementia [2]. Delirium and dementia are reported in the literature as parts of the continuum of postoperative cognitive impairment [3].

Delirium characterizes the following: (1) a disturbance of consciousness with inattention; (2) acute changes in cognition (i.e., memory deficits, disorientation, language disturbances, and perceptual disturbances); (3) the disturbances that develop over a short period of time and fluctuate over time; and (4) the disturbance is not caused by a general medical condition [4].

In the terminology relating to time course, delirium can be prevalent, incident, or persisting. Motor subtypes are classified into hyperactive delirium (characterized by increased psychomotor activity with agitation), hypoactive or “quiet” delirium (with reduced psychomotor behavior and lethargy), and mixed delirium, which alternates between a hyperactive and hypoactive manifestation. Additional definitions include subsyndromal delirium or delirium superimposed on dementia [2, 4, 5]. POCD is the subtle impairment of memory, concentration, and information processing [2, 4]. The symptoms of POCD vary from mild memory loss to the inability to concentrate or process information [2]. The nature of postoperative cognitive disorders is frequently subclinical and no changes in diagnostic imaging are present [6]. Therefore, in many cases, only the patient and/or partner can recognize the onset of the pathology [2].

In clinical practice, postoperative delirium is diagnosed by the Confusion Assessment Method (CAM). This method assesses four features: (1) acute onset and fluctuating course, (2) inattention, (3) disorganized thinking, and (4) altered level of consciousness [2]. The diagnosis of delirium requires the presence of the first two features and either third or fourth [2, 5]. In the diagnosis and grading of delirium, several tests are validated, including the CAM, the Delirium Rating Scale Revised-98, the Delirium Symptom Interview, the NEECHAM Confusion Scale, and the Estimation of Psychologic Ability and Surgical Stress (E-PASS) [5]. However, there are no approved criteria for the assessment and diagnosis of POCD [2, 5]. Therefore, POCD is much more difficult to define. There are three types of POCD through which patients can suffer from isolated learning/memory decline, difficulties in executive functions, or combined cognitive decline [7]. The diagnosis of POCD requires perioperative neuropsychological testing. Several tests are used, such as the Logical Memory Test, the CERAD word list memory, the Boston Naming Test, the Category Fluency Test, the Digit Span Test, the Trail Making test, and the Digit Symbol Substitution test [5]. Common diagnostic criteria include a 20% change from the baseline evaluation and a predefined (usually two or more) number of tests or an absolute decline (>1 SD) from baseline scores in two or more psychological tests [4].

Variability in the incidence of POCD can be caused by variable test batteries, the nonstandardization of neuropsychological tests performed at different times of the day, the lack of a control group, differences in significance levels between studies, significant loss of patients during follow-up as well, and the so-called “learning effect,” which occurs when the same test is applied to the same person many times [2, 27]. Another question concerns the time at which the diagnosis of POCD was made. Different drugs administered in the perioperative period can affect patients' cognition. Thus, some authors believe that the diagnosis of POCD should be made no earlier than two weeks after the surgery [27].

Delirium is the manifestation of cortical dysfunction resulting from disturbances in neurotransmitter systems. Abnormal serum anticholinergic activity as well as melatonin, norepinephrine, and lymphokines was described in the etiology of delirium. A relationship with surgical stress and inflammatory response was also suggested [5]. The risk factors of postoperative delirium include advancing age (>70), sensory deprivation (visual or hearing impairment), sleep deprivation, social isolation, physical restraint, use of urinary bladder catheter, iatrogenic adverse events, polypharmacy, preoperative use of opioids or benzodiazepines, severe illness (especially infection, fracture, or stroke), cognitive impairment, previous history of delirium or cognitive impairment, decreased cerebral perfusion pressure, fever or hypothermia, dehydration, malnutrition, low serum albumin, and a serum urea nitrogen/creatinine ratio of 18 or greater. Significant blood loss during surgery, blood transfusion, postoperative hematocrit <30%, and severe postoperative pain also were identified as a risk factors of postoperative delirium [2, 5].

According to Monk and Price [2], increasing age, lower education level, a history of a previous cerebral vascular accident with no residual impairment, and POCD at hospital discharge are identified as independent risk factors for POCD three months after surgery. Other studies included the following factors for the risk postoperative cognitive impairment: a general anesthesia rather than a regional one (although some authors [5] did not found it to be significant), increasing duration of anesthesia, reoperation, postoperative infections, postoperative respiratory complications, lower preoperative level of consciousness, and treatment with cholinergic drugs and benzodiazepines. Additional risk factors are noise, bright light, and physiologic disturbances, such as hyponatremia or hypoalbuminemia, as well as male sex, depression, and reduced activity in daily life [27–32]. Surprisingly, there was no evidence that hypoxemia is associated with the development of POCD [30, 33]. Some studies found that hypotension was the only intraoperative risk factor responsible for postoperative delirium [31]. However, other authors did not support that observation [30].

The incidence of postoperative delirium varies from 5% to 15%. In some patients, such as those with hip fracture, the problem is common and varies from 16% to 62% [5]. The incidence of POCD is difficult to describe. According to Deiner and Silverstein [5], it should be described at specific intervals after surgery; between the second and tenth day after surgery, the incidence is as much as 25%. The incidence then decreases as follows: to 10% at three months, 5% at six months, and 1% at one year [5]. According to Coburn et al., the incidence of POCD one week after surgery in patients older than 18 years varies from 19% to 41%, and a rate of 10% three months after surgery is detected in patients older than 60 years [34]. In 60-year-old patients who underwent major surgical procedures under general anesthesia lasting over two hours, 10% suffered memory impairment and concentration problems for more than three months after the surgery. The disorder occurred twice as often in patients between 70 and 80 years than in patients between 60 and 70 years [35]. According to statistical data, about 70% of patients with POCD die within five years, compared to about 35% of patients without postoperative delirium [36].

## 2. Surgery-Induced Stress Response and the Role of Anesthetic Agents in Neuroinflammation

Surgery-induced stress response leads to the following: (1) the cardiovascular effects of tachycardia and hypertension resulting from the increased secretion of catecholamines from the adrenal medulla and norepinephrine from the presynaptic nerve terminals because of the activation of the sympathetic nervous system; (2) changes in hormone secretion in hypothalamic-pituitary-adrenal axis, which influences the metabolism of carbohydrates, proteins, fat, salt, and water; and (3) immunological and hematological changes [37]. Immunological and hematological changes include cytokine production, acute phase reaction, neutrophil leukocytosis, and lymphocyte proliferation. Cytokines—mainly interleukin-1 (IL-1), tumor necrosis factor-*α* (TNF-*α*), and IL-6, which are released from activated leukocytes, fibroblasts, and endothelial cells—play an important role in systemic inflammatory reaction [37]. Some authors [5, 15, 27, 32, 38–41] suggest that inflammation plays a substantial role in the pathogenesis of POCD. Rudolph et al. [42] found that the chemokine concentration in the early postoperative period was more elevated in patients who developed delirium, compared to the matched controls. However, Lemstra et al. [38], in comparison to 18 patients who developed postoperative delirium with 50 controls, found no differences in preoperative concentrations of C-reactive protein, IL-6, and insulin growth factor 1 (IGF-1) between groups. Animal studies also showed that the development of POCD in rats was associated with glial activation and the expression of proinflammatory cytokines within the hippocampal region [27]. Some studies revealed the role of interleukin-18 (IL-18) in the neuroinflammation and neurodegeneration of the central nervous system. Patients with a defect in the IL-18 cytokine promoter gene had higher concentrations of serum amyloid peptides [36].

The increased production of TNF-*α*, IL-*β*, and IL-6 in mice neurons after isoflurane anesthesia was described by Wu et al. [43]. However, Schilling et al. found that volatile anesthetics (especially sevoflurane and desflurane) reduced proinflammatory cytokine release [44]. The exact mechanism by which volatile anesthetics increase proinflammatory cytokines remains unknown. It was suggested that nuclear factor kappa B-dependent (NF-*κ*B-dependent) pathways and the receptor for advanced glycation end products (RAGE) play a role in this mechanism [39].

The amyloid-*β* peptide concentration was related to learning, memory deficiencies, and neurodegeneration. The continuous infusion of amyloid-*β* peptide in rats resulted in impairments in learning and memory. Higher levels of amyloid-*β* peptide in the hippocampus were observed in older rats, compared to younger rats [36].

The literature includes a discussion on the role of anesthesia in the development of postoperative cognitive decline. However, the mechanism of the association of POCD with surgery and anesthesia remains unclear. However, some theories consider these effects of anesthetics, which include direct toxicity, alterations in calcium homeostasis, the systemic inflammatory effect, the age-sensitive suppression of neuronal stem cell function, and the acceleration of endogenous neurodegenerative processes, as well as caspase activation and apoptosis [45, 46]. Cell culture studies have shown that volatile anesthetics (isoflurane, sevoflurane, and desflurane, the latter in the presence of hypoxia) induce apoptosis and increase amyloid-*β* formation [36, 45]. It was shown that isoflurane is an agent promoting *β*-site amyloid precursor protein-cleaving enzyme (BACE) activity and amyloid-*β* deposition [39]. In an animal model, Dong et al. found that that sevoflurane increased BACE concentration and amyloid-*β* [47]. In addition, halothane produced the concentration-dependent enhancement of amyloid-*β* oligomerization [39]. Moreover, many anesthetics can promote the hyperphosphorylation of the microtubule-associated protein Tau, which was observed in hypothermic conditions, but not normothermic conditions [36, 45]. Propofol increased Tau phosphorylation, even with normothermia [48]. Fodale et al. [36] found that intravenous anesthetics, such as propofol and thiopental, did not significantly change the amyloid precursor protein [36].

Some authors discussed the role of genetic factors in the pathogenesis of neurodegenerative disorders. They found an association between the apolipoprotein *ε*4 (APO-*ε*4) allele and Alzheimer's disease [27]. Hence, the APO-*ε*4 gene could be a predictor of postoperative cognitive disorders [36]. However, in Abildstrom et al.'s study of 976 patients aged 40 years and older and undergoing noncardiac surgery, the *ε*4 allele was found in 272 patients. No significant association was found between the *ε*4 genotype and POCD [49].

## 3. POCD in Orthopedic Patients

According to Scott et al. [50], the incidence of POCD after big joint arthroplasty varies from 16% to 45%, although it was reported [51] as high as 72% at six days and 30% at six months, postoperatively. The etiology of POCD in orthopedic patients is unclear. Many factors are posited, including thromboembolic complications, the influence of anesthesia, and the influence of pain therapy in the postoperative period [50]. The high incidence of cognitive dysfunction in orthopedic patients can result (in addition to the above-mentioned risk factors) from long bone fractures, from prolonged immobilization, and partially from perioperative stress [6] or a surgery technique. Colonna et al. concluded that the incidence of cerebral embolization after lower extremity arthroplasty was between 40 and 60% [52]. Fatal cerebral embolization constituted complications accompanying long bone fractures [53], total knee replacements [54], hip arthroplasty [55], and vertebroplasty [56], in which the embolic material passed into the brain through an open foramen ovale [57], although postmortem examinations did not reveal it [52].

## 4. Biomarkers of Brain Damage

Biochemical tests are useful diagnostic tools in the examination of functional brain disorders. Elevated serum concentrations of the markers of brain damage indicate a neuronal and/or glial injury. The biomarkers are released because of either transient ischemia or ultimate cell degradation. Their serum concentration depends on the localization of pathological changes, the degree of tissue damage, and the time that has passed since the onset of changes. The ideal marker of brain damage should have the following characteristics: (1) highly specific; (2) highly sensitive; (3) released in only cases of irreversible damage to cerebral neurons; (4) detectable in the blood and/or cerebrospinal fluid within a short period of time after the injury; and (5) released in well-known time sequences after the injury. Furthermore, the marker should be (6) age- and sex-independent and (7) easily detectable in the blood because frequent drawing of cerebrospinal fluid samples is impractical; and (8) its concentration should be easily measureable in laboratory tests [58].

The following substances have been investigated as relevant neurological biomarkers in the postoperative period.

### 4.1. S100B Protein

The S100B protein has a molecular weight of 21 kDa. It belongs to the calcium-mediated proteins in the S100 proteins family, which consists of 24 members that have similar structures and functions [59]. Some members of the S100 protein family are specific for certain localizations [60]. High S100B protein concentrations are present inside the brain, mainly in astroglial and Schwann cells, as well as in adipocytes, chondrocytes, and melanocytes [10, 61, 62]. The S100B protein plays different roles in the human body and is present in many types of cells and tissues [63]. It has intra- and extracellular targets, and it has autocrine and paracrine effects on glia, neurons, and microglia [64]. Although the exact functions of the S100B protein are still unclear, it may be involved in neuronal and glial growth, proliferation, and activation [64].

Increased S100B concentrations in serum and cerebrospinal fluid were observed after brain infarction, trauma, and toxic injury [64]. The highest S100B protein serum level was observed just after an injury [58] and was then normalized within 24 hours, even in patients with poor outcomes [65]. An increased concentration of the S100B protein was shown on the sixth day after head trauma, which was probably the result of a secondary injury [65]. Elevated concentrations of S100B protein were also demonstrated in a posttraumatic animal model [65]. The results of animal studies suggested that S100B protein levels correlated with the degree of shock: in moderate shock, they were higher than in severe shock [66]. The concentration of S100B protein increased immediately after bilateral long bone fractures, as well as after local ischemia and the reperfusion of the liver, gut, and kidneys [10, 66]. In rabbits with femur fracture and no evidence of neurological injury, S100B protein concentration increased within minutes after bone trauma, suggesting that the S100B protein was released from nonneuronal sources [64]. This finding supports observations that elevated levels of the S100B protein can be caused by increased permeability of the blood-brain barrier, regardless of cerebral damage [65].

Elevated levels of S100B protein were shown in basketball and hockey players after competition, as well as in runners, boxers [67], swimmers, and soccer players. In the latter, however, latter there was a correlation between increased protein concentration and frequency of head injury [13]. Intense physical exercise can remarkably increase serum S100B concentration. It was shown that, after acute muscle injury, S100B expressed in mature muscle myofibrils was released from injured muscle tissue and could penetrate the bloodstream [68]. Another possible cause of increased serum S100B level is the catecholamine-dependent activation of adipocytes [68].

A raised plasma level of the S100B protein also occurred in melanoma patients [69] and in sepsis-associated encephalopathy [70].

The possibility that the S100B protein could be released from extracerebral localization restricts its utility as a marker of brain damage, which, nonetheless, still ranges from 70 to 80% [62]. The S100B protein is a very useful biochemical tool because of its short (25 minutes) half-life [58, 62, 64], as well as the fact that its serum concentrations are not affected by age or sex [58]. Moreover, serum concentrations are not altered by alcohol overdose, moderate renal dysfunction, or hemolysis [62, 64].

### 4.2. Neuron Specific Enolase (NSE)

Neuron specific enolase (NSE) is an enzyme that catalyzes the conversion of 2-phospho-D-glycerate to phosphoenolpyruvate in a glycolytic pathway. It is found in the cytoplasm of neurons and neuroendocrine cells and its subunits, and *α* and *γ* are specific for neurons. NSE is also found in red cells and platelets [64, 71]. The molecular weight of NSE is 78 kDa, and its half-life is 24 hours [64]. The normal serum concentration of NSE varies between 2 and 20 mg/L; values >30 mg/L are pathological, and ≥115 mg/L are related to poor prognosis [64]. Increased levels of NSE were observed after cortical brain injury and severe head trauma and in patients with temporal lobe epilepsy as well as in the patients with internal cardiac defibrillators, where correlations were found between NSE levels and the number of shocks and the cumulative time of cardiac arrest [64].

This protein is effective in predicting neurological outcomes after cardiac arrest and in patients with ischemic stroke [64]. The results of a study on cardiac surgery patients were ambiguous. In some studies, the correlation of NSE with POCD was observed [12, 20], but not in others [11]. The NSE concentration in the cerebrospinal fluid in patients after aortic aneurysm repair surgery was increased regardless of the presence or absence of neurological symptoms [64]. Gempp et al.'s study of recreational divers found that NSE levels >15.9 *μ*g/L predicted the development of neurological decompression sickness with a specificity of 100% [26]. Observations of patients undergoing liver transplantation revealed that the postreperfusion concentration of NSE correlated with decreased regional oxygen saturation [64].

Similarly, the results of studies analyzing the correlation between the APOE4 genotype and cognition with NSE serum levels in the postoperative period were inconclusive [64].

### 4.3. Glial Fibrillary Acidic Protein (GFAP)

The glial fibrillary acidic protein (GFAP) is a monomeric filament protein found in the astroglial skeleton [71]. It is a specific marker of brain damage and is potentially useful in predicting clinical outcomes [71]. It was shown that the serum GFAP levels were higher in patients with mass lesions than in those with diffuse brain injury [71]. Pelinka et al. [72, 73] found that serum GFAP levels were increased in patients with intracranial pressure (ICP) above 25 mmHg. However, the cutoff value of GFAP for detection ICP elevation was not defined.

Clinical data suggests that GFAP provides important information for the prognosis of traumatic brain injury as well as for differential diagnosis and prognosis in various types of stroke [74]. The most recent data showed that, in intracerebral hemorrhage, GFAP sensitivity is 0.8 and specificity is 0.97 [75].

### 4.4. Tau Protein

The Tau protein is a microtubule-associated protein that stabilizes the axonal microtubules [64]. It is found in the brain and the spinal cord [64]. The tau protein has different isoforms, with molecular weight varying from 45 to 68 kDa [64]. The phosphorylation of the Tau protein is associated with neuronal death, and it is observed in neurodegenerative diseases [64]. Increased concentrations of the tau protein were observed in patients who developed postoperative cognitive decline [64]. A correlation between increased serum levels of the tau protein and the size of brain infarction was observed in noncardiac surgery patients [64].

### 4.5. Metalloproteinases (MMP)

Metalloproteinases (MMP) are zinc-dependent endopeptidases that degrade most extracellular matrix proteins [64]. MMPs are secreted as cells in blood vessel walls as myocytes, endothelial cells, and macrophages [64].

MMPs are divided into the following: (a) gelatinases (MMP-2, MMP-9); (b) collagenases (MMP-1, MMP-8, and MMP-13); (c) matrilysins (MMP-7); (d) membrane-type MMPs (MMP-14, MMP-15, MMP-16, and MMP-17); and (e) others (MMP-11, MMP-12) [64].

MMP-9 was observed to be a marker of blood-brain barrier dysfunction, and elevated concentrations of this protein were noted in patients with stroke [64]. Gaudet et al. [16], in their study of 73 patients undergoing carotid surgery, found elevated serum concentrations of MMP-9 in patients who developed postoperative cognitive decline. This finding is supported by Taurino et al., who found that, in patients undergoing carotid surgery, MMP-9 levels were significantly higher in those with cerebral lesions at neuroimaging, compared to the healthy controls [14].

### 4.6. Ubiquitin C Terminal Hydrolase-L1 (UCH-L1)

Ubiquitin C terminal hydrolase-L1 (UCH-L1), also called neuronal-specific gene product (PGP 9.3), is a highly specific neuronal protein with a molecular weight of 24 kDa. It is found in perikarya in gray matter. Its role is removing excessive, oxidized, or misfolded proteins in the central nervous system [64, 71].

Because it is not found in nonneuronal sources, UCH-L1 is a specific biomarker of brain lesions [64]. After traumatic brain injury and subarachnoid hemorrhage, this protein is released to CSF [64]. In Papa et al.'s study, which includes 96 patients with traumatic brain injury and 199 controls, UCH-L1 was detectable in serum within one hour after the injury and was associated with GCS scoring, CT lesions, and the need for neurosurgical intervention [22].

### 4.7. Microtubule-Associated Protein 2 (MAP2)

Microtubule-associated protein 2 (MAP2) is thought to be a dendrite-specific protein and, according to some authors, it is a good biomarker for dendritic injury [71]. In Mondello et al., MAP2 concentrations correlated with the Glasgow Outcome Scale Extended (GOSE) and the Levels of Cognitive Functioning Scale (LCFS), measured at six months after injury [21].

### 4.8. Myelin Basic Protein (MBP)

Myelin basic protein (MBP) represents 30% of the protein content of myelin. The protein consists of four isoforms with molecular weights ranging from 14 to 21.5 kDa. Changes in MBP concentrations were observed after cortex contusion in an animal model. However, there is no evidence for the use of MBP as a biomarker after traumatic brain injury or in intracranial hypertension in humans [71].

### 4.9. *α*-II Spectrin Breakdown Products (SBDP) 150, 145, and 120

The *α*-II spectrin protein forms part of the axolemmal cytoskeleton, stabilizes the structure of myelinated axons, and is a major substrate in calpain-1 and calpain-2 and caspase-3, which are involved in cellular necrosis and apoptosis [71]. The calpain-specific 150- and 145-kDa SBDPs are used as biomarkers of necrotic neuronal death, and the caspase-3-specific 120-kDa SBDP is used as a biomarker of apoptosis [71]. In study of 40 adult patients with traumatic brain injury, mean CSF concentrations of SBDP-145 and SBDP-120 were higher in patients with brain injury who died than in those who survived. SBDP-145 levels >6 ng/mL and SBDP-120 levels >17.55 ng/mL strongly predicted death. The authors concluded that SBDP-145 seems more accurate in predicting outcomes in patients suffering from traumatic brain injury [17].

### 4.10. Micro-RNA (miRNA)

Micro-RNA (miRNA) particles are small molecules involved with regulation of gene expression [70]. MiRNA plays an important role in orthopedic diseases, such as osteoarthritis and rheumatoid arthritis [76]. Changes in microRNA concentrations were observed in patients with bone tumors [77]. The comparison of patients with severe brain injury and healthy volunteers revealed that decreases in the levels of miR-16 and miR-92a and increased levels of miR-765 were strong markers of severe traumatic brain injury at 25 to 48 hours after injury [71].

## 5. Biomarkers of Brain Damage in Orthopedic Patients

Increased levels of biomarkers were observed after orthopedic procedures and bone fractures.

Kinoshita et al. [9] examined 14 patients, half of which underwent total knee arthroplasty (TKA) with bone cement. The other half underwent intramedullary nail stabilization of the tibia. All procedures were performed with tourniquet and ischemia. In the TKA group, in blood samples withdrawn 15 minutes after tourniquet release, there was a statistically significant elevation of S100B serum level in comparison to the group in which tibial fracture was stabilized with an intramedullary nail. The authors suggested that the increase was caused by the transient injury of brain tissues caused by the bone cement [9].

Tomaszewski et al. examined changes in S100B protein levels in patients who underwent total hip arthroplasty, with or without bone cement. In both groups of patients, the mean preoperative concentration of S100B protein was comparable to that in the healthy subjects and reached the maximum just after the operation. In the cemented group, the level was significantly higher than in the noncement group, and normalization was slower. Elevated serum S100B protein levels may be due to the release of S100B protein from bone marrow, as well as the transfer of cellular materials from the site of the surgery through the bloodstream into the brain. Because all patients with intraoperative mean blood pressure dropping below 50 mmHg were excluded from the study, hypotension was eliminated as a possible cause of the elevation of S100B concentration [18].

In their study of 83 patients older than 65 years undergoing elective total hip arthroplasty, Ji et al. analyzed the perioperative concentration of the Tau protein, the phosphorylated Tau protein (pTau), 42 amino acids in the form of amyloid *β*1 (A*β*1-42), Tau/A*β*1-42, pTau/A*β*1-42, brain-derived neurotropic factor (BDNF), IL-6, IL-1*β*, C-reactive protein (CRP), and malondialdehyde (MDA). They found that patients who developed POCD had significantly higher levels of IL-1*β*, Tau/A*β*1-42 ratio, and pTau/A*β*1-42 ratio and the lower level of A*β*1-42 in the cerebrospinal fluid, compared to the non-POCD group. There was no difference in CFS levels of tau protein, pTau, BDNF, and IL-6 between both groups. The authors concluded that such biomarkers might predispose the development of POCD in aged patients after hip replacement surgery under spinal anesthesia [23]. Similarly, Xie et al.'s study involving 136 patients who had total knee or hip replacement surgery found that preoperative CSF A*β*40/Tau and A*β*42/Tau ratios were associated with the postoperative scores of neurocognitive tests. Therefore, the A*β*/Tau ratio may identify patients with a higher risk of POCD development [24].

However, increased serum concentration of the S100B protein was observed after injuries, which did not include brain damage. The highest levels of the S100B protein were noted in patients with long bone fractures [8]. Studies conducted on patients with isolated bone fractures without brain injury revealed that patients with hip, radius, or tibia fractures had significantly higher concentrations of the S100B protein, but those with phalange, hand, or foot fractures did not [78]. Animal studies showed increased S100B serum levels after bilateral femur fractures in rats; these results indicated that bone marrow could be a potential source of the S100B protein [10]. Increased serum levels of the S100B protein were found in patients with acute spinal fracture, but without head injury [79]. Observations of 233 patients after trauma found that the highest concentrations of S100B (1.68 *μ*g/L on admission and 0.31 *μ*g/L after 6 hours) were noted in patients suffering from multitrauma with head injury. There were no differences in the S100B levels in patients with or without isolated head injury (0.47 and 0.14 *μ*g/L and 0.49 and 0.15 *μ*g/L, resp.) [80].

van Munster et al. analyzed the serum concentrations of S100B protein and NSE in 120 patients with hip fracture and a mean age of 83.9 years. Sixty-two patients experienced delirium. The authors observed a difference between the levels of S100B, but not NSE, in the first samples taken during delirium and the samples from nondelirious patients [15]. The simultaneous comparison of cortisol, IL-6, IL-8, and S100B protein revealed that the highest levels of cortisol and IL-8 were observed before delirium but the highest levels of IL-6 and S100B were observed during delirium. In multivariable analysis, cortisol, logIL-6, and logS100B were associated with delirium, but when adjusted for preexisting cognitive impairment, only logS100B remained associated [81]. With the exception of the previously cited work of van Munster et al., NSE concentration and delirium in orthopedic patients have received scant attention in the literature published in English.

Anckarsäter analyzed the perioperative levels of five CSF biomarkers: the Tau protein, pTau protein, A*β*42, neurofilament light (NFL), and GFAP in 35 patients undergoing knee arthroplasty under regional blockade. CSF Tau and GFAP concentrations increased, whereas pTau, A*β*42, and NFL were unchanged. CSF Tau and pTau significantly correlated with the CSF/serum albumin ratio as an indicator of blood-brain barrier permeability. The CSF Tau protein concentration also correlated with the administered doses of bupivacaine [25]. Witlox et al.'s study of 66 older adults with hip fracture found no differences in preoperative CSF A*β*1-42, Tau protein, and pTau in patients who did and did not develop delirium during hospitalization [19]. Summary of clinical studies on biomarkers of brain damage and their relation to orthopedic surgery and/or postoperative cognitive disorders was shown in Table 1.

## 6. Possible Explanation of Increased Concentration of Biomarkers of Brain Damage in Orthopedic Patients

The relationship between stress response, neuroinflammation, and biomarkers was previously mentioned. The question of surgical technique is also important. During some orthopedic procedures, bone cement is used to fix the elements of the implants to the bone base. However, it has been shown that the use of cement can lead to hemodynamic instability, a decrease in cardiac output, heart contractility, systemic vascular resistance, and blood pressure, that is, the so-called bone cement implantation syndrome [82]. Hemodynamic changes affect cerebral perfusion, as in the relation between S100B concentration and degree of shock [66]. Although the presence of bone cement inside the medullar cavity itself did not produce hypotension, hemodynamic instability was often observed when the prosthesis stem into the bone is hammered into the bone [83] and when the pressure inside the marrow cavity increases. The higher the pressure, the better the penetration of the cement into the bone and the greater the strength of osteosynthesis. However, because of the increased pressure, the translocation of the cellular material at the site of the surgery into the systemic circulation was facilitated, causing this material to reach the lungs via the bloodstream. The diameter of the lung capillaries is about 8 *μ*m. In 1956, Niden and Aviado showed the possibility of transferring glass spheres up to 420 *μ*m in diameter through the pulmonary vessels [84]. The second method used to circumvent the pulmonary filter was via the foramen ovale, which in one-third of the population is closed only functionally. Thus, embolic material could be transferred into the brain. In ultrasound examination the presence of cellular material from the site of the surgery in the circulatory system is shown by as a “snow flurry.” In Hayakawa et al. [85], the “snow flurry” was observed from the beginning of the reaming of the femoral canal until the end of the surgery, and it intensified while the cemented prosthesis stem was being inserted into the bone. This was not noted during procedures that did not use bone cement. A histological examination of the elements forming the “snow flurry” revealed the presence of amorphous eosinophilic particles with fibrin attached to their surface. The same effect was noted in patients who had undergone cemented hip arthroplasty during the whole procedure, either before or after the use of bone cement. Fat particles or bone marrow was not detected in any sample; the authors thought that they had examined “bone dust” particles with attached fibrin fibers [85]. The work was limited to a relatively small group of only seven patients. In Kim et al., the histological examination of samples from the right atrium revealed the presence of fat particles in 34% and 44% of procedures and the presence of bone marrow cells in 13% and 11% of procedures, with and without bone cement, respectively [86]. The contribution of bone cement to the etiology of thromboembolic events was suggested [56]. Clark et al. showed a transient, but statistically significant, decrease of cardiac output by 33% and of stroke volume by 44% during procedures that used bone cement. Before the use of bone cement, there were no changes between the two (i.e., with and without cement) groups. Because embolic material was released either before or after the use of bone cement, the decrease in cardiac output and stroke volume might have been followed by the embolic material originating from bone marrow or vasodilatation caused by a monomer [87]. An animal studies with dog showed that an intravenous injection of the acrylic acid monomer did not affect the partial pressures of oxygen and carbon dioxide in the arterial blood. Mild and transient hypotension was observed, while monomer concentration in the pulmonary artery was much higher than that noted in usual clinical situations [88].

It should be noted that discussing the role of bone cement only in increased concentrations of biomarkers and the etiology of POCD is an oversimplification. The role of advanced age in the patients, their comorbidities, disturbances in blood flow (because of such problems such as atrial fibrillation and immobilization), and the consequences of long bone fractures can lead to the increased incidence of thromboembolic events, as described above.

Another issue concerns the effect of previously prescribed medications, the duration of hospitalization, the role of administered pharmacotherapy, and the impact of the anesthetic procedure on the development of postoperative cognitive decline.

## 7. Biomarkers of Brain Damage, Delirium, POCD, and Anesthetic Procedures

Previous research has considered the influence of different types of anesthesia (general versus regional) on elderly patients. Previously published clinical studies did not show the prevalence of any anesthesia in POCD prevention [6]. Evered et al. compared the incidence of POCD in 644 patients who underwent coronary angiography under sedation, total hip replacement surgery under general anesthesia, and coronary artery bypass graft surgery (CABG) under general anesthesia to 34 subjects in control group. The authors observed a higher incidence of POCD in elderly patients at day 7 after CABG than after the orthopedic procedure. However, the POCD at three months after the operation was independent of the type of surgery and anesthesia used [89]. However, a systematic review of Zywiel et al. on the influence of anesthesia and pain management on cognitive dysfunction after joint arthroplasty suggested that general anesthesia might be associated with increased risk of postoperative cognitive decline in the early postoperative period, compared to regional anesthesia, although the effect was not seen beyond seven days [90]. Some predisposing and precipitating factors associated with delirium and/or POCD, such as age, preexisting cognitive impairment, severe illness, anemia, immobilization, decreased oral intake, dehydration, sleep deprivation, and urinary catheter use, were common in all patients, regardless of the type of anesthesia. Regional anesthesia can be induced with a lower number of drugs, compared to general anesthesia. Moreover, the pain control is better and the incidence of thromboembolic complications is lower. However, the contraindications to neuroaxial blockade include disturbances in coagulation and circulatory failure. Thus, a number of orthopedic procedures on the lower limbs are performed under general anesthesia.

Neurotransmitters take part in the regulation of conscience, memory, and learning through the central cholinergic system [36]. Hence, the interactions between anesthetic drugs and this system may be important in the pathogenesis and development of POCD. There are two main classes of cholinergic receptors: nicotinic and muscarinic. Nicotinic acetylcholine receptors (nAChRs) are ligand-gated cation channels, and muscarinic acetylcholine receptors (mAChRs) are ligand-gated K+ channels, which are divided into five subtypes (M1–M5). The agonists of central mAChRs and nAChRs may improve, while the antagonists could impair performance in cognition, learning, and memory [36].

Volatile anesthetics and ketamine are potent inhibitors of nAChRs. Desflurane selectively binds the M1 subtype. Sevoflurane depresses the M1 and M2 subtypes, whereas isoflurane interferes only with the M3 subtype. All barbiturates are competitive antagonists of mAChRs. Propofol acts on mAChRs and nAChRs, but in concentrations higher than those used clinically do. Fentanyl and morphine inhibit signals mediated by both types of receptors, and remifentanil does not change the release of acetylcholine from cholinergic nerves. Furthermore, neuromuscular blocking agents or neostigmine administered during general anesthesia can influence cholinergic transmission [36].

Another question concerns the possible neurotoxicity of general anesthetics. The findings of both cell-culture and animal studies suggest that anesthetics may cause neuroapoptosis, caspase activation, neurodegeneration, *β*-amyloid protein accumulation, and oligomerization, leading to deficits in cognition. It has been shown that desflurane has a less harmful neurotoxic profile compared to other volatile anesthetics [46]. This finding was supported by Zhang et al. [91], who observed that the administration of isoflurane, but not desflurane, was associated with an increase in human CSF amyloid-*β* 40 concentrations at 24 hours after anesthesia, compared to the values observed in patients under spinal anesthesia. Desflurane, but not isoflurane, was associated with the decrease in amyloid-*β* 42 levels at two hours after anesthesia. In this study, both isoflurane and desflurane did not significantly affect the concentration of the tau protein in human CSF [91].

Hudetz et al. [92, 93] found that a single administration of the intravenous anesthetic ketamine at 0.5 mg/kg during the induction of anesthesia reduced the incidence of POCD to one week after cardiac surgery. The authors concluded that the anti-inflammatory properties of ketamine produced this result.

The results of two studies [94, 95] analyzing the influence of the multimodal anesthetic technique on the incidence of POCD were conflicting. However, methodological inconsistencies obscured the clear interpretation of the results [90].

### 7.1. Postoperative Pain Management Strategy and Postoperative Cognitive Decline

Effective postoperative pain management in the postoperative period minimizes the use of opioids, which can decrease the incidence of postoperative cognitive decline [90]. Zywiel et al. [90] cited 12 studies that analyzed the influence of different postoperative pain management strategies on the risk of POCD. Langford et al. [96], in their study of 525 patients who underwent major noncardiac surgery, found decreased incidence of POCD on the second day after surgery (1.8% versus 5%), with the intravenous administration of parecoxib, compared to the placebo. Marino et al. [97] investigated the efficacy of continuous lumbar or femoral block, and YaDeau et al. [98] described the efficacy of single-shot femoral nerve block after TKA. These regional techniques decreased the incidence of POCD in orthopedic patients. Interestingly, the intra-articular administration of bupivacaine did not change the incidence of POCD after TKA, compared to the placebo [90].

The postoperative use of opioids is associated with a higher risk of development of POCD, regardless of parenteral drug administration (intravenous, intramuscular, or epidural). The use of morphine, compared to fentanyl, was associated with the increased risk of POCD, when opioids were administered either intravenously [99, 100] or epidurally [101]. It also was found that the intravenous administration of opioids was associated with the higher incidence of POCD, compared to oral drug administration [90].

## 8. Presurgery Neuroanatomical Biomarkers for Postoperative Cognitive Decline

Previous studies have focused on the evaluation of brain damage markers in the postoperative period and their correlation with postoperative cognitive decline. However, of greater importance is the identification of the predictive factors of postoperative cognitive disorders amongst patients scheduled for surgery. It is known that certain cerebral regions, such as the entorhinal cortex (ERC) and the hippocampus, may change with Alzheimer disease. Leukoaraiosis and lacunae volume may indicate vulnerability for postoperative executive dysfunction [102]. Price et al. [102] analyzed the hypothesis that presurgical neuroanatomical markers, such as MRI-based hippocampus/ERC and leukoaraiosis/lacunae volume, may predict cognitive changes in the postoperative period. Their findings suggested that the value of presurgery ERC/hippocampal volumes as neuroanatomical predictors for cognitive decline was limited. However, perioperative leukoaraiosis and lacunae volume, as neuroimaging evidence of microvascular disease, helped explain postoperative executive function decline.

The results are preliminary as yet; nevertheless, these observations are interesting and should be a stimulus for further research.

## 9. Optimization of Surgical and Anesthetic Procedures in the Prevention of POCD

A fast-track set-up reduces the duration of hospitalization. Krenk et al. showed that when the length of stay of patients who underwent hip and knee arthroplasty was reduced from 7 to 10 days, with a median of 3 days, no cases of postoperative delirium were observed in the analyzed population [103]. In a series of 225 patients over 60 years, no cases of postoperative delirium were observed, and the incidence of POCD was reduced by more than 50% at one week, postoperatively [104]. The most recent data confirmed the above observations [105].

It is important to achieve the proper level of anesthesia during surgery. As mentioned previously, surgery-induced stress response has unwanted cardiovascular, metabolic, and immunological effects. On the other hand, overly deep levels of anesthesia may decrease cardiac function and organ perfusion. Therefore, the question of the potential neurotoxicity of general anesthetics remains unanswered. Farag et al. analyzed patients under general anesthesia; those with lower values on the Bispectral Index (BIS) had fewer disturbances in cognitive functions, especially in information processing, between the fourth and sixth weeks after surgery [106]. However, this observation seems isolated. Steinmetz et al. analyzed 70 patients with cerebral state index monitoring (CSI) and found no significant association between deep (CSI < 40) and light (CSI > 60) anesthesia [107]. Chan et al. found that BIS-guided anesthesia reduced anesthetic exposure and decreased the risk of POCD at three months after surgery. The authors concluded that when the depth of anesthesia is maintained at BIS values from 40 to 60, for every 1,000 patients undergoing major surgery, 23 were prevented from POCD and 83 were prevented from delirium [108]. BIS-guided anesthesia also improved the outcomes of surgical procedures [109].

During noncardiac surgery, significant cerebral desaturation occurred in up to 30% of patients [109]. Papadopoulos et al. described the association of cognitive dysfunction in elderly patients with hip fractures and low values of cerebral oxygenation [110]. Thus, the monitoring of cerebral oxygen saturation may be promising in the reduction of subtle neurologic deficits [111], particularly in patients who undergo total hip arthroplasty [112]. However, in a systematic review of cardiac surgery patients, Zheng et al. suggested that data are insufficient to conclude that interventions to improve cerebral regional saturation prevent stroke or POCD [113].

## 10. Summary

Although cognitive dysfunction in hospitalized patients is important both clinically and socially, it is difficult to analyze methodologically. Disturbances at the cellular level can manifest as mood disorders and lead to deterioration in patients' functioning and social assessment. It is very difficult to define either the normal state or the pathology of cognitive functions.

Because of the increase in age at hospitalization as well as in the number of orthopedic procedures, the issue of postoperative cognitive decline is gaining importance. A previous review on POCD and brain damage markers following large joint arthroplasty was published in 2011 [114]. Since then, knowledge on the biomarkers of brain damage and their correlation with cognitive impairment is becoming much more detailed. Studies on the utility of the different substances as potential biomarkers are being performed. The results of studies on cerebral oxygen saturation measured by near-red spectroscopy and its correlation with postoperative cognitive decline carry great promise. Despite the need for further tests, it is now known that patients at risk of postoperative cognitive disorders should be identified before surgery. From this perspective, the results of studies on neuroanatomical biomarkers will likely have future clinical applications.

The identification of patients with preexisting risk factors for POCD, shortening the period of time preceding the surgery, the appropriate technique used in the procedure, and adequate intraoperative monitoring as well as physical and intellectual exercises, nutrition, and medication play important roles in decreasing the incidence of neurocognitive deficits in the elderly.

## Figures and Tables

**Table 1:** Summary of clinical studies on biomarkers of brain damage and their relation to orthopedic surgery and/or postoperative cognitive disorders.

Author(s)	Study design	Results	Conclusions	Reference
Anderson et al., 2001	Analysis of serum S100B concentrations for a normal population (*n* = 459) and multitrauma patients without head injury (*n* = 17).	The mean serum S100B concentration for a normal healthy population was 0.032 *μ*g/L. Among trauma patients, serum S100B levels were highest after bone fractures and thoracic contusions. Burns and minor bruises also produced increased S100B levels.	Trauma, even in the absence of head trauma, results in high serum concentrations of S100B. S100B may have a negative predictive value to exclude brain tissue damage after trauma.	[8]

Kinoshita et al., 2003	Patients (*n* = 14) undergoing TKA with bone cement use (*n* = 7) or reamed intramedullary nailing for tibial fracture (*n* = 7).	The serum level of S100B was increased after a pneumatic tourniquet deflation in the TKA group compared with the tibial fracture group.	In patients undergoing TKA, bone cement may transiently induce astroglial injury, although it does not alter neurological outcomes.	[9]

Pelinka et al., 2003	Bilateral femur fracture in 10 anesthetized rats.	S100B concentration was increased after bilateral femur fracture and reached a peak 30–120 minutes after fracture.	S100B is increased after bilateral femur fracture without hemorrhagic shock in rats.	[10]

Stolz et al., 2004	Patients (*n* = 37) undergoing aortic valve replacement.	New DWI lesions (14 patients, 3 with focal deficits) correlated with age, preexisting T2 lesion volume, and postoperative S100B concentrations after surgery. In a forward stepwise canonical discrimination model, only T2 lesion volume was a relevant variable.	The volume of preexisting T2 lesions is related to the development of perioperative DWI lesions.	[11]

Ramlawi et al., 2006	Patients (*n* = 40) undergoing cardiac surgery under CPB.	The incidence of NCD was 40%. Both NSE and tau protein were elevated in the presence of NCD compared with those without NCD. S100B increase was not different between the NCD and control patients. Cardiotomy suction increased S100B levels; NSE and tau were not influenced.	NSE and tau are better associated with NCD and less influenced by cardiotomy suction compared with S100B.	[12]

Stålnacke et al., 2006	Female soccer players (*n* = 44) before and after a competition.	Concentration of both S100B and NSE was increased after the game, with correlation between S100 concentration and both the number of head injuries and other trauma events.	S100B and NSE were increased by game activities. The increases in S100B concentration were related to the number of head injuries and other trauma events.	[13]

Taurino et al., 2008	Patients (*n* = 15) undergoing carotid endarteriectomy.	MMP-9 levels were higher in patients with carotid stenosis versus controls, significantly in those with cerebral lesions at neuroimaging.	MMP-9 assay could be useful in the evaluation of carotid lesions to help identify those at highest risk of a neurologic event.	[14]

van Munster et al., 2009	Patients (*n* = 120) aged 65 years or more with hip fracture.	The incidence of delirium was 51.7%. Delirious state, pre- or postoperative status, and type of fracture were associated with S100B levels. The highest S100B levels were found “during” delirium. No difference in S100B or NSE levels was seen regardless of subtype of delirium.	Delirium was associated with increased level of S100B.	[15]

Gaudet et al., 2010	Patients (*n* = 73) undergoing carotid endarteriectomy.	Approximately 19% of eligible patients developed NCD. Compared to patients without NCD, this group had both higher total and activity MMP-9 levels at baseline.		[16]

Mondello et al., 2010	Adult patients (*n* = 40) with severe TBI who underwent ventriculostomy.	Mean CSF levels of SBDPs were higher in TBI patients than in controls. SBDP145 provided accurate diagnoses at all time-points examined, while SBDP120 release was more accurate 24 h after injury. Within 24 h after injury, SBDP145 CSF levels correlated with GCS scores, while SBDP120 levels correlated with age. SBDP levels were higher in patients who died than in those who survived. SBDP145 levels (>6 ng/mL) and SBDP120 levels (>17.55 ng/mL) strongly predicted death.	CSF SBDP levels can predict injury severity and mortality after severe TBI and can be useful complements to clinical assessment.	[17]

Tomaszewski et al., 2010	Patients (*n* = 60) undergoing THA with (*n* = 30) or without (*n* = 30) bone cement use.	Following surgery, the S100B levels were increased in both groups. However, S100B concentration in the cement group was higher and its normalization was slower, in comparison to the noncement group. No clear changes in neuropsychological tests between both groups were observed.	There was a relationship between bone cement implantation and elevated S100B postoperatively; however, neuropsychological test results did not reflect this.	[18]

Witlox et al., 2011	Participants (*n* = 77) aged 75 and older admitted for surgical repair of acute hip fracture.	Postoperative delirium occurred in 39.5%. Preoperative CSF A*β*1-42, tau, and P-Tau levels were not different between participants who did and did not develop delirium.	CSF markers for plaque and tangle formation are not strongly associated with delirium risk in older adults with hip fracture.	[19]

Jones et al., 2012	Participants (*n* = 68) over 60 years old following major surgery.	Baseline NSE and the change in NSE levels between baseline and 24 h were correlated with the change in CAMCOG score between baseline and 52 weeks.	NSE may be a useful predictor of individuals at risk of more severe long-term cognitive decline.	[20]

Mondello et al., 2012	Patients (*n* = 16) with severe TBI (GCS ≤ 8) 6 months after injury and in 16 controls.	Severe TBI patients had higher serum MAP-2 concentrations than controls with no history of TBI at 6 months after injury. MAP-2 levels correlated with the GOSE and LCFS at month 6. Lower serum levels of MAP-2 were observed in VS patients compared to non-VS patients.	Severe TBI results in a chronic release of MAP-2 in patients with higher levels of consciousness, suggesting that remodeling of synaptic junctions and neuroplasticity processes occur several months after injury. The data indicate MAP-2 as a potential marker for emergence to higher levels of cognitive function.	[21]

Papa et al., 2012	Adult patients (*n* = 96) with blunt head trauma.	Mean UCH-L1 levels in patients with positive CT scans were higher in comparison to those with negative CT.	UCH-L1 is detectable in serum within an hour of injury and is associated with measures of injury severity including the GCS score, CT lesions, and NSI.	[22]

Ji et al., 2013	Patients (*n* = 83) older than 65 years undergoing elective THA.	POCD occurred in 24.6% at 7 days after surgery. Patients with POCD had significantly higher IL-1*β*, Tau/A*β*1-42, P-Tau/A*β*1-42, and a lower level of A*β*1-42 in CSF when compared with the non-POCD group. There were no differences in preoperative CSF levels of Tau, IL-6, and P-Tau as well as plasma levels of IL-1*β*, IL-6, BDNF, and CRP between POCD and non-POCD groups.	The POCD patients were associated with higher postoperative plasma levels of MDA and higher IL-1*β* and lower A*β*1-42 levels in preoperative CSF that might predispose the development of POCD in aged patients following THA with spinal anesthesia.	[23]

Xie et al., 2013	Patients (*n* = 136) undergoing THA/TKA.	Preoperative CSF A*β*-42/tau ratio was associated with postoperative Hopkins Verbal Learning Test Retention and the Benton Judgment of Line Orientation. A*β*-40/tau ratio was associated with Brief Visuospatial Memory Test Total Recall.	Preoperative CSF A*β*/tau ratio is associated with postoperative changes. The presence of biomarkers, specifically the A*β*/tau ratio, may identify patients at higher risk for cognitive changes after surgery.	[24]

Anckarsäter et al., 2014	Patients (*n* = 35) undergoing TKA under spinal anesthesia.	CSF T-Tau concentrations increased during and after surgery and were correlated with the administered doses of bupivacaine. P-Tau, A*β*-42, and NFL remained unchanged, while the mean GFAP level increased with a large standard deviation. CSF T-Tau and P-Tau correlated with the CSF/serum albumin ratios.	Bupivacaine may be involved in impaired cortical axonal integrity during nonneurological surgery.	[25]

Gempp et al., 2014	Divers (*n* = 59) with neurological DCS and 37 asymptomatic divers.	NSE, but not S100B protein, was higher in the DCS group than in controls.	NSE was found to be useful for the diagnosis of neurological DCS. Reliability of S100B was not demonstrated.	[26]

BNDF: brain-derived neurotrophic factor; CAMCOG score: Cambridge Assessment for Mental Disorder in the Elderly; CPB: cardiopulmonary bypass; CRP: C reactive protein; CSF: cerebrospinal fluid; CT: computer tomography; DCS: decompression sickness; DWI: diffusion-weighted imaging; GCS: Glasgow Coma Scale; GFAP: glial fibrillary acidic protein; GOSE: Glasgow Outcome Scale; LCFS: Level of Cognitive Function Scale; NCD: neurocognitive decline; NFL: neurofilament light; NSE: neuron-specific enolase; NSI: neurosurgical intervention; POCD: postoperative cognitive dysfunction; SBDPs: *α*II-spectrin breakdown products; TBI: traumatic brain injury; THA: total hip arthroplasty; TKA: total knee arthroplasty; UCH: ubiquitin C-terminal hydrolase; VS: vegetative state.
